# Virus-host interactomics: new insights and opportunities for antiviral drug discovery

**DOI:** 10.1186/s13073-014-0115-1

**Published:** 2014-11-29

**Authors:** Benoît de Chassey, Laurène Meyniel-Schicklin, Jacky Vonderscher, Patrice André, Vincent Lotteau

**Affiliations:** ENYO Pharma SAS, Lyon, 69007 France; Hospices Civils de Lyon, Lyon, France; CIRI, Université de Lyon, Lyon, 69365 France; Inserm, U1111, Lyon, 69365 France

## Abstract

The current therapeutic arsenal against viral infections remains limited, with often poor efficacy and incomplete coverage, and appears inadequate to face the emergence of drug resistance. Our understanding of viral biology and pathophysiology and our ability to develop a more effective antiviral arsenal would greatly benefit from a more comprehensive picture of the events that lead to viral replication and associated symptoms. Towards this goal, the construction of virus-host interactomes is instrumental, mainly relying on the assumption that a viral infection at the cellular level can be viewed as a number of perturbations introduced into the host protein network when viral proteins make new connections and disrupt existing ones. Here, we review advances in interactomic approaches for viral infections, focusing on high-throughput screening (HTS) technologies and on the generation of high-quality datasets. We show how these are already beginning to offer intriguing perspectives in terms of virus-host cell biology and the control of cellular functions, and we conclude by offering a summary of the current situation regarding the potential development of host-oriented antiviral therapeutics.

## Introduction

Conventional drug therapies against human viruses mainly target viral enzymes (Table 1). The repertoire of druggable viral proteins and corresponding small molecules is extremely limited, and a major drawback in the use of these direct-acting drugs is the emergence of resistance [1-3]. Because of these limitations, antiviral drug discovery is beginning to explore the possibility to develop host-oriented molecules acting on cellular functions that are essential for viruses to replicate [4]. Indeed, viruses are obligate intracellular parasites, and, as such, they rely on cellular functions to replicate. They have evolved a variety of strategies to manipulate the cellular machinery for their own benefit as well as to counteract or even to use host immune defenses. As the vast majority of cellular functions is supported by interacting proteins, the manipulation of cellular processes by viruses mainly results from physical interactions between viral and host proteins [5]. Therefore, a virus-host (VH) interactome, interpreted in the context of the host interactome, allows the identification of a network of cellular proteins and associated functions that are essential in the virus life-cycle. These proteins can be considered as new antiviral targets, and some of them could well be functionally manipulated with new small molecules, repurposed drugs (Food and Drug Administration (FDA)-approved or experimental molecules) or with rescued drugs from abandoned pharmaceutical pipelines [4,6-9].

**Table 1:** **Current FDA-approved antivirals and their targets**

**DrugBank ID**	**Name**	**Type**	**Year of first approval as an antiviral**	**Virus**	**Target(s)**
DB00249	Idoxuridine	Small molecule	1963	HSV	DNA, viral thymidine kinase
DB00915	Amantadine	Small molecule	1966	Influenza virus	Viral matrix protein M2
DB00987	Cytarabine	Small molecule	1969	Herpesviruses	Human cytidine deaminase, human cytochrome P450 3A4, human deoxycytidine kinase, human 5’-nucleotidase, human deoxycytidylate deaminase
DB00194	Vidarabine	Small molecule	1976	HSV, VZV	Viral DNA polymerase, viral thymidine kinase, DNA
DB00811	Ribavirin	Small molecule	1980	HCV, RSV	Human inosine-5’-monophosphate dehydrogenase 1, human adenosine kinase, human cytosolic purine 5’-nucleotidase
DB00787	Aciclovir	Small molecule	1982	HSV1, HSV2, VZV	Viral DNA polymerase, viral thymidine kinase
DB00495	Zidovudine	Small molecule	1987	HIV	Viral reverse transcriptase
DB01004	Ganciclovir	Small molecule	1989	CMV	Viral DNA polymerase, viral thymidine kinase, DNA
-	Tromantadine	Small molecule	Later than 1990	HSV	Human glycoproteins
-	Interferons	Proteins	1990s	Hepatitis, etc.	Human IFNARs
DB00900	Didanosine	Small molecule	1991	HIV	Viral reverse transcriptase
DB00529	Foscarnet	Small molecule	1991	CMV, HSV	Viral DNA polymerase
DB00943	Zalcitabine	Small molecule	1992	HIV	Viral reverse transcriptase
DB00426	Famciclovir	Small molecule	1994	HSV, VZV	Viral DNA polymerase
DB00478	Rimantadine	Small molecule	1994	Influenza virus	Viral matrix protein M2
DB00649	Stavudine	Small molecule	1994	HIV	Viral reverse transcriptase
DB00709	Lamivudine	Small molecule	1995	HIV, HBV	Viral reverse transcriptase
DB00432	Trifluridine	Small molecule	1995	HSV	Viral thymidylate kinase
DB00577	Valaciclovir	Small molecule	1995	HSV, VZV, CMV	Viral DNA polymerase, viral thymidine kinase
DB00369	Cidofovir	Small molecule	1996	CMV	Viral DNA polymerase
DB00224	Indinavir	Small molecule	1996	HIV	Viral protease
DB00238	Nevirapine	Small molecule	1996	HIV	Viral reverse transcriptase
DB00299	Penciclovir	Small molecule	1996	HSV	Viral DNA polymerase, viral thymidine kinase
DB00503	Ritonavir	Small molecule	1996	HIV	Viral protease
DB01232	Saquinavir	Small molecule	1996	HIV	Viral protease
DB00705	Delavirdine	Small molecule	1997	HIV	Viral reverse transcriptase
DB00220	Nelfinavir	Small molecule	1997	HIV	Viral protease
DB01048	Abacavir	Small molecule	1998	HIV	Viral reverse transcriptase
DB00625	Efavirenz	Small molecule	1998	HIV	Viral reverse transcriptase
-	Fomivirsen	Oligonucleotide	1998	CMV	Viral mRNA
DB00110	Palivizumab	Humanized monoclonal antibody	1998	RSV	Viral fusion glycoprotein F0
DB00701	Amprenavir	Small molecule	1999	HIV	Viral protease
DB00198	Oseltamivir	Small molecule	1999	Influenza virus	Viral neuraminidase
DB00558	Zanamivir	Small molecule	1999	Influenza virus	Viral neuraminidase
DB00632	Docosanol	Small molecule	2000	HSV	Viral envelope glycoprotein
DB01601	Lopinavir	Small molecules	2000	HIV	Viral protease
DB00022	Peginterferon alfa-2b	Protein	2001	HCV	Human IFNARs
DB00300	Tenofovir	Small molecule	2001	HIV, HBV	Viral DNA
DB01610	Valganciclovir	Small molecule	2001	CMV	DNA
DB00718	Adefovir Dipivoxil	Small molecule	2002	HBV	Viral DNA polymerase
DB00008	Peginterferon alfa-2a	Protein	2002	Hepatitis	Human IFNARs
DB01072	Atazanavir	Small molecule	2003	HIV	Viral protease
DB00879	Emtricitabine	Small molecule	2003	HIV	Viral reverse transcriptase
DB00109	Enfuvirtide	Protein	2003	HIV	Viral envelope glycoprotein
DB01319	Fosamprenavir	Small molecule	2003	HIV	Viral protease
DB00442	Entecavir	Small molecule	2005	HBV	DNA
DB00932	Tipranavir	Small molecule	2005	HIV	Viral protease
DB01264	Darunavir	Small molecule	2006	HIV	Viral protease
DB01265	Telbivudine	Small molecule	2006	HBV	Viral DNA polymerase, DNA
DB04835	Maraviroc	Small molecule	2007	HIV	Human CCR5
DB06817	Raltegravir	Small molecule	2007	HIV	Viral integrase
DB06414	Etravirine	Small molecule	2008	HIV	Viral reverse transcriptase
DB08873	Boceprevir	Small molecule	2011	HCV	Viral NS3 protein
DB08864	Rilpivirine	Small molecule	2011	HIV	Viral reverse transcriptase
DB05521	Telaprevir	Small molecule	2011	HCV	Virus NS3-4A protease

*Abbreviations*: *CMV* cytomegalovirus, *HBV* hepatitis B virus, *HCV* hepatitis C virus, *HSV* herpes simplex virus, *IFNAR* interferon alpha/beta receptor, *RSV* respiratory syncytial virus, *VZV* varicella zoster virus.

Until 2007, VH protein-protein interactions (PPIs) had been explored with low-scale experiments focusing on a particular viral protein or a specific biological process. The recent application of high-throughput screening (HTS) methods to the establishment of VH interactomes has not only greatly enriched the landscape of VH PPI but has also yielded an explosion in candidate drug targets. Furthermore, substantial efforts have been made to integrate both low- and high-throughput data in various databases (Table 2), favoring the transition from a reductionist to an integrative approach to understanding viral infection.

**Table 2:** **Databases of virus-host protein-protein interactions and drug-targets**

***VH PPI databases***				
**Name (reference)**	**Description**	**VH interactions**		**Search date**
**IntAct/MINT** ** [** 87 **]**	Open-data molecular-interaction database populated by data curated either from the literature or from direct data depositions	5,717 (query performed through IMEx single entry-point)		July 2014
**DIP** ** [** 88 **]**	Database that catalogs experimentally determined protein interactions that are either curated or computationally extracted			
**Uniprot** **[** 89 **]**	Protein sequence reference database. Among numerous annotations are listed some binary protein interactions quality-filtered from IntAct			
**VirusMentha** ** [** 51 **]**	Resource that specifically captures and presents interactions between viral and host proteins curated by databases that are part of the IMEx consortium	5,846		October 2014
**VirHostNet** ** [** 52 **]**	Knowledge base dedicated to literature- and database-curated interactions between viral and human proteins	3,113		July 2014
***Drug-target databases***				
**Name (reference)**	**Description**	**Drugs**	**Targets**	**Search date**
**DrugBank** **[** 90 **]**	High-quality knowledgebase. Provides extensive information on drugs, their mechanisms of action and their associations with targets	7,739	4,092	July 2014
**Therapeutic Target Database** **[** 91 **]**	Conceptually similar to DrugBank. Provides links between primary therapeutic targets and their corresponding drugs	20,667	2,360	July 2014
**ChEMBL** **[** 92 **]**	Large-scale database dedicated to the description of biological activities of numerous chemical entities with drug-like properties, manually curated from the medicinal chemistry literature	1,359,508	9,414	July 2014

*Abbreviations*: *IMEx* International Molecular Exchange, *PPI* protein-protein interaction, *VH* virus-host.

Altogether, the wealth of VH PPI data has already provided access to nearly complete interactomes for several viruses that are of public health concern, including influenza virus, hepatitis C virus (HCV) and dengue virus [10]. Integration of this information into knowledge of the uninfected human protein network highlights key topological and functional features of the ‘infected network’. High-throughput approaches also allow comparative analyses, such as virulence factors versus other factors [11] and oncogenic versus non-oncogenic factors [12-14], and the differential targeting of crucial intracellular pathways [15,16].

One successful FDA-approved host-targeting antiviral drug is Maraviroc, a CCR5 chemokine receptor antagonist for the treatment of HIV infection [17] (Table 1). Other antivirals are being designed to target viral receptors, but a challenging and promising strategy is the use of pre-existing small molecules to drug intracellular interactors of viral proteins that have been initially designed to treat other diseases. Considering the exponentially growing number of candidate cellular targets from interactome studies, such drug repositioning is becoming a potentially more efficient way to increase the therapeutic antiviral arsenal.

Here, we will review and discuss recent advances in approaches for high-throughput VH PPI screening and the implications of these recent findings for understanding the landscape of VH PPI. We will describe the main insights for basic research as well as the potential for antiviral drug discovery. Finally, we feature some examples of promising and successful antiviral molecules targeting host proteins.

## Approaches for high-throughput screening of virus-host protein-protein interactions

**Figure 1:**
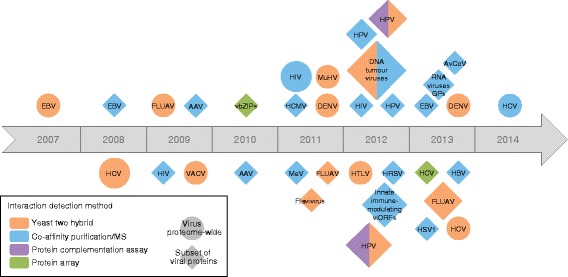
**Timeline of studies describing the results of virus-host protein-protein interactions high-throughput screens.** Circles indicate virus proteome-wide screens. Diamonds show studies of a particular subset of viral proteins. The various colors indicate the type of detection method used. The size of each shape is approximately proportional to the number of VH PPIs detected. Abbreviations: AAV, adeno-associated virus; CMV, cytomegalovirus; DENV, dengue virus; EBV, Epstein Barr virus; FLUAV, influenza A virus; GP, glycoprotein; HBV, hepatitis B virus; HCV, hepatitis C virus; HIV, human immunodeficiency virus; HRSV, human respiratory syncytial virus; HSV1, herpes simplex virus 1; HPV; human papillomavirus; HTLV, human T-lymphotropic virus; ORF, open reading frame; RSV, respiratory syncytial virus; VACV, vaccinia virus; VZV, varicella zoster virus.

Since the first descriptions of VH protein interactions in the late 1980s, the associated methodologies have been adapted to large-scale studies. Yeast two-hybrid (Y2H) and co-affinity purification remain the most frequently used technologies, while protein arrays and protein-complementation assays are emerging as promising approaches. As high-throughput data production does not have a universally accepted definition, we have chosen to review technologies that have generated more than 100 VH PPIs. Using this definition, 35 reports can be referred to as HTS of VH PPIs since 2007 (Figure 1).

**Figure 2:**
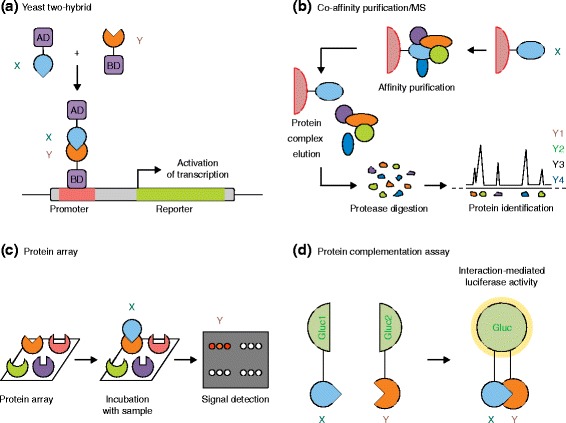
**Methods used for high-throughput screening of virus-host protein-protein interactions. (a)** The yeast two-hybrid approach. The generic principle of a Y2H system is based on the reconstitution of a functional transcription factor following interaction between a bait protein and a prey protein. One construct comprises the DNA-binding domain of the transcription factor (BD) in fusion with a bait protein, whereas the prey protein is fused with the transcription activation domain (AD). Upon interaction of the bait with the prey in the nucleus of the yeast, the transcription factor activity is reconstituted, leading to the transcription of a reporter gene. In general, reporter genes are selected for their ability to allow the growth of yeast on selective medium or the use of a colorimetric assay so that their active transcription can be easily monitored. Bait and prey interactions can be tested pairwise in an array when both baits and preys have been individually cloned or upon the screening of fusion proteins expressed from cDNA libraries followed by sequencing of selected preys. **(b)** The co-affinity purification/MS technique. This approach is typically divided into two technical steps consisting of the capture of cellular proteins with the bait protein and identification of affinity-purified proteins by mass spectrometry (MS; method reviewed in [86]). **(c)** The protein array. Functional protein arrays, also called ‘protein chips’, can comprise a thousand different proteins attached at high density on a solid surface [30]. Following binding of a protein of interest with its target, the interaction can be detected with fluorescent, radioisotope or photochemical tags. **(d)** Protein-complementation assays. These assays employ a split *Gaussia princeps* luciferase (Gluc) assay together with bait and prey proteins that are expressed in mammalian cells in fusion with two inactive fragments of the luciferase. Interaction between bait and prey brings the two fragments into close proximity, restoring the enzymatic activity.

Since the pioneering description of the Y2H approach in 1989 by Fields and Song [18], Y2H and its various technological improvements have been among the methods of choice for the construction of VH interactomes (Figure 2a). The first two unbiased genome-wide VH PPI screens using Y2H technology were performed for Epstein-Barr virus and HCV. These studies relied on an initial construction of a viral ORFeome, comprising cloned open reading frames (ORFs) encoding a complete set of viral proteins, and led to the identification, respectively, of 173 and 314 VH PPIs [19,20]. The Y2H technology has been used in 15 high-throughput screens since these founding studies, for viral genome-wide interactome exploration or for focusing on a subset of viral proteins (Figure 1). Construction of viral and human ORFeome collections and implementation of versatile recombinational cloning systems (such as Gateway (Life Technologies, Gaithersburg, MD, USA)) are essential tools that have allowed this approach to become particularly powerful. For example, Shapira and colleagues [21] tested the interactions between the 10 influenza virus proteins and 12,000 human proteins available in the human ORFeome v3.1 [22]. The versatility of the Gateway system allows easy transfer of cDNAs into any compatible expression system for further interaction or functional studies. The ViralORFeome database was constructed to provide the scientific community with an integrated set of bioinformatics tools enabling the potential capture of viral ORFs in the Gateway recombinational cloning system and to make available a collection of viral cDNAs in Gateway-compatible plasmids [23]. Nevertheless, interactions discovered using Y2H screens must be confirmed by a secondary method, such as co-affinity purification, to reduce the risk of false-positive interactions and to increase the confidence in the dataset, which is usually expected to reach >80% [20]. The problem of false-negative interactions is more difficult to address - the sensitivity of this technology does not exceed 25% [24], so that repetitive samplings of the same search space are mandatory to reach completeness.

While Y2H screens tend to detect transient binary interactions, co-affinity purification coupled to mass spectrometry (coAP/MS) assays aim at detecting stable complexes [25], exploring overlapping and complementary interaction search spaces (Figure 2b). One major strength of this method, compared with Y2H, is that it can be performed under more-physiological conditions, allowing context-dependent identification of interactions. The tandem affinity purification (TAP) technique is a variation of co-affinity purification that is characterized by a lower contaminating background [26]. The TAP strategy involves the use of two tags and two sequential steps of affinity purification. This method has been used to generate the largest numbers of VH PPI data, for the targeting of host proteins by viral immune modulators [27] and by tumor virus proteins [13] that identified, respectively, 1,681 and 3,787 VH protein associations (Figure 1).

Protein array technologies emerged in 2010 as a promising approach to study VH PPI (Figures 1 and 2c). In a first screen, an original array was printed with human and viral leucine zipper regions of 33 human basic leucine zipper domain proteins and four viral proteins. By probing with fluorescently labeled versions of the same proteins, 101 interactions were detected [28]. This approach was well validated by circular dichroism (CD) spectroscopy that determines whether there are changes in the conformation of proteins when they interact. Use of CD confirmed all the retested interactions. A second screen performed in 2013 used a commercial human protein microarray kit containing 9,000 human proteins that identified 100 interactions with the HCV core protein as a probe [29]. This technology is rapidly evolving to improve sensitivity, to increase proteome coverage and to allow the development of label-free optical tools and the quantification of the association-dissociation rate of protein interactions in a high-throughput format [30]. More recently, HTS of VH PPI by using a protein complementation assay has been implemented by Jacob and coworkers (Figures 1 and 2d) [12,14]. Comparative VH interactomes were explored for E2, E6 and E7 proteins from a range of pathogenic and non-pathogenic human papillomaviruses. Benchmarking this method with random protein pairs and a positive reference set confirmed the performance of this assay in a high-throughput setting [31].

Because the presence of false positives and false negatives is inherent to HTS, quality control of the datasets is a major issue. Multiple approaches have been developed for the Y2H strategies, including the diversification of reporter genes, low plasmid copy number and retesting hits by subcloning ORFs into fresh yeast [11,32-34]**,** that have greatly helped to improve the quality of the datasets. A database of cDNAs considered to be false positives for the classic Y2H system is also available as a work in progress [35] thanks to the work of Golemis and co-workers [36]. In a related attempt, last year the CRAPome database, a repository of common contaminants in coAP/MS experiments, was constructed to allow better characterization of background associated with this technology (for example, proteins that bind to the bead matrix used during the precipitation, antibodies conjugated to the beads or the epitope tag) [37]. Recent technical improvements also contributed to lower the rate of contaminants, and one of these techniques is known as ‘stable isotope labeling with amino acids in cell culture’ (SILAC) [38] coupled to co-affinity purification. SILAC is a powerful tool to discriminate background from specific interactions. Cells expressing the protein of interest and control cells are labeled with different non-radioactive isotopes (heavy (H) and light (L)). The quantification of the H:L ratio of proteins co-purified with the bait protein allows the relative quantification of recovered proteins. Nonspecific binding leads to a ratio of 1, whereas a high ratio indicates a possible specific interaction. This method has been successfully applied to the interactomic mapping of the nucleocapsid protein from highly pathogenic North American porcine reproductive and respiratory syndrome virus [39], the human respiratory syncytial virus NS1 protein [40], the coronavirus infectious bronchitis nucleocapsid protein [41], the HIV1 Gag protein [42], NS3 and NS5 proteins of dengue virus type 2 [43], and NS1 and NS2 proteins of influenza A virus [44].

These approaches are complementary and allow the exploration of different interaction search spaces. Other methods have also been developed to be amenable to a high-throughput format. Among them, MAPPIT is a cytokine-based mammalian PPI trap assay [45] and LUMIER is a tag-precipitation assay coupled to renilla luciferase [46]. To our knowledge, none of these methods has yet been applied in a high-throughput VH PPI study.

## Access to the comprehensive landscape of viral human protein targets

Systems biology and reductionist approaches are complementary to build a comprehensive landscape of viral infection and replication. High-throughput screening has revealed a large number of VH PPIs, and numerous studies have also provided detailed and often mechanistically oriented information on specific VH interactions. Therefore, it is a challenge to identify the wealth of VH PPI data that are available in the literature. Several databases have been developed to capture and structure these data, either through text mining or through manual curation [47]. The International Molecular Exchange (IMEx) consortium can be considered the key public curator of such data, focusing on manually curated data to ensure the high-quality datasets that are required for further analysis [48]. Created in 2005, this international collaboration framework now coordinates most of the major public-interaction data providers. They share the literature curation workload, applying high-level quality standards and provide the scientific community with unique access to the data [48]. The IMEx strategy limits redundancies as well as inconsistencies and improves curation coverage. IMEx partners have adopted a common curation policy that entails the use of the controlled vocabularies and formats first standardized by the Human Proteome Organization (HUPO) Proteomics Standards Initiative - Molecular Interaction (PSI-MI) working group in 2002 [49].

VH PPIs are represented by nearly 6,000 non-redundant physical interactions highlighted by searching the available databases (IntAct/MINT, DIP and Uniprot, searched between July and October 2014; Table 2). VH PPIs are also accessible in VirusMentha, an iteration of the interactome browser mentha that presents non-redundant virus-related interactions extracted from manually curated PPI databases that have adhered to the requirements of the IMEX consortium [50,51] (Table 2). Finally, the VirHostNet database also offers a high-quality dataset of approximately 3,100 curated VH PPIs but has not been updated since 2009 [52] (Table 2).

Additional efforts to construct a clean repository of VH PPIs have been made but are difficult to trace because they often result from isolated initiatives. For several years, we have been performing our own manual curation of VH PPIs in the literature, according to PSI-MI standards. From our own experience, this is a highly demanding task, especially when it comes to viruses for which species, strains and protein identifiers have to be clearly defined, and because mature proteins are often not identified in viral polyprotein sequences. Papers with large datasets are also often difficult to process because of their inconvenient format and because of the heterogeneity in protein-annotation systems.

**Figure 3:**
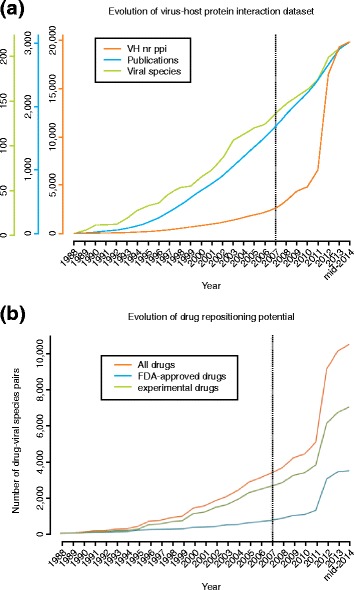
**Virus-host protein-protein interaction dataset and drug-repositioning potential. (a)** Evolution of the VH PPI dataset over the past 26 years. Orange indicates the number of non-redundant VH PPIs; blue shows the number of publications describing at least one VH PPI; and green gives the number of viral species for which at least one VH PPI has been described (source: PubMed). **(b)** Evolution of drug-repositioning potential over the same time-scale as in **(a)**. Number of drug-viral species combinations inferred from the VH PPI dataset. Orange shows all drugs; blue shows FDA-approved drugs only; and green indicates experimental drugs only.

The number of publications describing VH PPIs is now over 3,000, involving more than 200 viral species (Figure 3a). The identification of non-redundant VH PPIs has been growing exponentially since 2007, with the use of HTS methods (Figures 1 and 3a). The accumulation of VH PPIs also might allow increased confidence in interactions that are redundantly described in the literature.

Despite efforts to gain confidence in HTS data, overlaps between VH PPI datasets are often very low. Experimental protocols are not yet standardized from lab to lab, from the choice of technology to differences in scoring cutoffs. For instance, if Y2H has been the most popular strategy so far to construct VH interactomes, technological variations of this generic approach are very important at different essential steps, such as the reporter genes, yeast strains, plasmid copy number, fusion proteins, stringency conditions and libraries, that have an obvious impact on the outcome of the experiment [53]. Another important consideration is the dynamic nature of many VH PPIs during the course of the infection. For instance, Sindbis virus nsP3 protein has been shown to interact with several heterogeneous nuclear ribonucleoproteins primarily at the early times of infection, whereas interactions with 14-3-3 epsilon, zeta and eta were only observed at later times during infection [53]. Sindbis virus nsP4 protein was found associated with five specific host factors at early times in infection and ten others at later times [54]. This highlights the importance of the physiological context evolving during the infection and that can also differ according to the type of cells and the conditions of infection. Independent of the technology, an important variable that could influence the overlap between VH screens is the heterogeneity of the virus protein sequences. This is mostly exemplified for RNA viruses, whose polymerases display a high mutation rate [55]. As a consequence, an RNA virus referred to as a primary isolate is not genetically homogeneous. The sequence of a viral protein can be highly divergent from the sequence of a reference protein, and this could be responsible for the loss or gain of interactions. Finally, some interactions might be missed owing to inherent limitations of the technologies that are used. For instance, Y2H is not compatible with membrane proteins or with self-activating proteins, and some interactions might require post-translational modifications from mammalian cells. Tags or reporter proteins that are fused with baits or preys can cause steric hindrance and prevent protein interactions. To gain confidence in a biophysical interaction, orthogonal validations using other interaction methods are therefore required so that a confidence score can be calculated [24]. After more than two decades of studying VH PPIs, the overlap of recent screens for the most-studied viruses with previous studies is now reaching 25% (HCV [56], influenza virus NS1 protein [44]). Bearing the above considerations in mind**,** it is possible that this rate of overlap defines a near-complete dataset of cellular proteins that are in interaction with an extensively studied virus.

VH interactomes are representative of which interactions might occur during the infection but do not unambiguously identify biologically relevant cellular targets before a functional validation of the interactions. The functional validation is mostly assessed by modulating the expression levels of cellular proteins (overexpression, knockout or knockdown). In a recent exploration of HCV-host PPIs, RNA interference screening of viral protein interactors revealed that 21.7% were essential for viral replication [56]. This rate of validation is in the range of previous work [11,21] and is well above the rates identified from genome-wide small interfering RNA screens (between 0.45% [57] and 1.36% [58]). This indicates that combining interactomics with functional genomics strongly enhances the biological relevance of a cellular protein for the replication of a virus. It should also be considered that, instead of modulating the quantity of a given cellular protein, anti-viral molecules will rather be designed to inhibit a catalytic cellular activity or to prevent a viral protein from interacting with one or several cellular partners. Therefore, although the combination of high-throughput strategies could help reduce the number of drug-target candidates in a funnel effect, a drawback is the possible emergence of false-negative targets and the exclusion of potentially interesting drug candidates.

## Recent insights from virus-human interactome studies

High-throughput screening studies of VH interactions were initially implemented to provide a comprehensive view of the interplay between a virus and its host. For example, mapping of the HCV infection protein network has shed new light on the molecular basis of the co-deregulation of insulin, Jak-STAT and transforming growth factor beta signaling pathways involved in the most frequent clinical syndromes, and it has identified the specific targeting of the focal adhesion pathway, thus providing new avenues for the study of tumor initiation and progression [20].

Other screens have been designed to identify the differential strategies exploited by closely related viruses to perturb the cellular network. Comparative interactomics of human papillomavirus E2 proteins clustered these proteins according to the pathogenic potential of the viral strains (high-risk versus low-risk), giving clues to the potential of therapies targeting specific proteins [14]. The TAP approach has been applied to profile the interactome of 70 viral immune modulators from 30 viral species, identifying an unexpected variety of cellular mechanisms exploited by individual viruses, families and groups [27]. Simultaneously, a systematic study of DNA VH interactomes (including papillomavirus, Epstein-Barr virus, adenovirus and polyomavirus, using both Y2H screens and TAP tag purifications) and transcriptome network perturbations revealed a rewiring of the cellular network and highlighted the Notch signaling pathway and deregulation of apoptosis in virus-induced cancer [13]. The first comparative mapping of interactions of a set of influenza A virus NS1 and NS2 proteins, chosen for their sequence diversity, revealed cellular targets involved in each step of the infectious process that are shared by all or the majority of the viral proteins [11].

Beyond the establishment of VH interactomes and the discovery of specific and common cellular functions targeted by viruses, studies have revealed the fundamental principles that have evolved by which viruses manipulate the cellular network [5,10,59,60]. Computational analysis of network-descriptive metrics (such as ‘degree’ and ‘betweenness’) raised striking observations regarding the centrality of viral targets in the context of the human protein network. Indeed, viral proteins showed a preferential interaction with high-degree cellular proteins - that is, proteins having a high number of direct interacting partners that are therefore locally highly connected in the human interactome. Viral proteins also have a strong tendency to interact with cellular proteins of high betweenness, which is a global centrality measure of the number of shortest paths that pass through a given protein and reflects the flux of information that is controlled by that protein. These topological characteristics of cellular proteins targeted by viral proteins have been observed from unbiased high-throughput VH interaction screenings and are indicative of the functional importance of these characteristics. Another general hallmark of viruses is that they can compensate for their small proteomes by the ability to interact with numerous cellular proteins. To allow this, they have evolved intrinsically disordered protein regions that are enriched for short linear motifs involved in multiple interactions in the human protein network [10,61]. Some of these motifs are adopted from the characteristics of their host by using a strategy of molecular mimicry (for example, the PDZ-binding motif at the carboxyl terminus of avian influenza NS1 proteins [62] and the polyproline motif on the HCV NS5A protein that is able to interact with Src-homology 3 (SH3) domains of cellular proteins [63]).

Taken together, proteomic analyses are boosting our knowledge of viral replication and disease etiology and are allowing the identification of new cellular targets that might be suitable for drug development.

## Advances in targeting viral interactors

### Antiviral drug discovery shifts towards host targets

The search for effective therapeutics to treat viral infections has been an active area of research for many years, resulting in both success and failure. Chronic infections by viruses such as HIV or hepatitis B virus (HBV) can now be controlled, but they require lifelong treatment. Treatments for acute viral infections - for example, by respiratory viruses or highly pathogenic emerging RNA viruses - are either poorly effective or do not exist. Overall, the treatment of viral infections largely remains an unmet medical need despite intense research activity. In addition to targeting viral components through direct-acting drugs (Table 1), recent efforts are now focusing on the identification of essential host factors as the targets of new antivirals. Targeting host factors dramatically enlarges the repertoire of therapeutic targets and offers a greater barrier to the emergence of resistance. Targeting host molecules has the potential for broad-spectrum indications when targeting pathways that are shared by the different variants of a given virus or by different types of virus [10]. Although far from complete, the construction of VH interactomes is starting to support this active field to identify the best cellular proteins to be targeted for an antiviral activity.

### Targeting human proteins

Antiviral small molecules that inhibit cellular functions or VH PPIs have been reported in the literature, but currently no database has been developed to reference them. Below, we review a selection of host-oriented molecules with antiviral activity *in vitro* or *in vivo* against two major viruses infecting humans, influenza and HCVs.

The antiviral market is worth more than US$4 billion and has a high growth rate. Recurrent seasonal influenza represents a significant part of this market, with 5 to 10% of the world population being infected each year by the influenza virus. A highly effective pan-strain vaccination remains the major objective to protect the population from this infection. Currently, protection relies on annual vaccination, offering variable and unpredictable efficacy, and on the antiviral neuraminidase inhibitors oseltamivir and zanamavir, which can be used for the treatment of established illness and for pre- and post-exposure prophylaxis in specific situations. However, the effectiveness of these drugs is strongly questioned, and the emergence of resistance and changes in seasonal and pandemic strains further decrease drug response. Because of the limited therapeutic options for epidemic and pandemic influenza, novel approaches to the development of influenza drugs are a public health priority.

**Figure 4:**
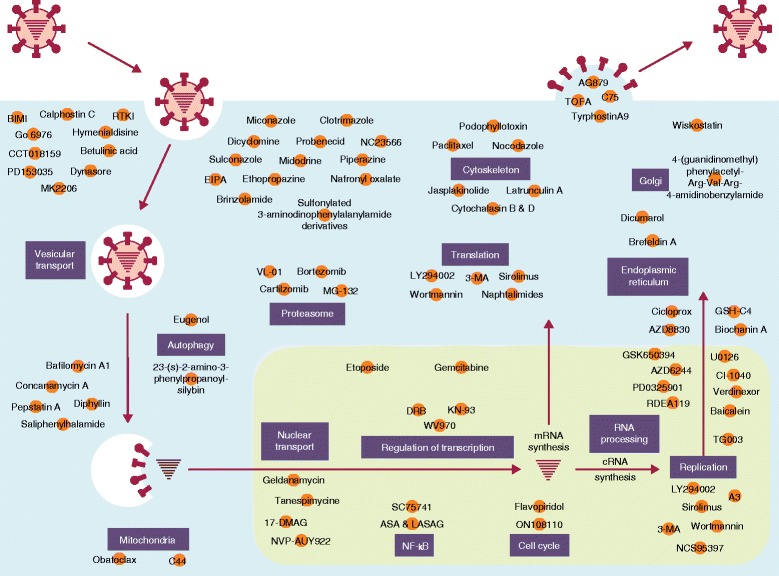
**Host-oriented molecules implicated in activities against influenza A virus replication.** Compounds targeting host proteins with an inhibitory impact on influenza virus replication have been positioned in the schematic according to their action on the virus life-cycle, when known, or else according to the subcellular localization of their target. None of these drugs, except LASAG, is currently being assessed in clinical trial as an anti-influenza virus drug.

Inhibiting influenza virus replication with drugs that target cellular proteins or cellular functions is now an established concept. Early studies first used these drugs for basic research [64,65], but, soon after, inhibitors of protein kinase C (PKC) and the Raf-MEK-ERK signaling cascades were tested for their therapeutic potential [66,67]. Since then, more than 80 compounds targeting host proteins have been identified for their inhibitory impact on influenza virus replication (Figure 4). These compounds target a large diversity of cellular proteins, acting at almost all steps of the virus replication cycle. Many of these inhibitory molecules were originally developed for anti-cancer indications and include agents such as MEK inhibitors [8], obatoclax and gemcitabine [68], flavopiridol [69], anti-cytoskeletal drugs [70] and etoposide [71], among others. Most of these drugs have an inherent toxicity when tested for long-term treatment, but it should be noted that treatment of severe influenza virus infections is not expected to last more than a few days. For treatment of non-severe influenza infections, additional molecules are actively being sought, and several extended interactomes that have identified more than 600 cellular targets of viral proteins are providing useful leads [11,21,44,51].

As mentioned above, a major problem in the use of direct-acting drugs for the treatment of viral infections is the high frequency of emergence of resistant strains. The development of host-targeted therapies is expected to reduce this risk. This has been tested experimentally by repetitive culture of influenza virus under pressure of direct-acting or host-oriented drugs. After five to ten passages, no reduction of the antiviral effect was observed using host-oriented molecules (a MEK inhibitor [72], inhibitors of NF-κB [3,73] or an inhibitor of Rac1 [74]), whereas the use of the direct-acting drugs oseltamivir or amantadine (the two classes of approved drugs for the treatment of influenza) led to rapid emergence of resistant variants. This indicates that the virus cannot easily adapt to a situation where cellular functions that are essential for its replication become less accessible and further suggests that targeting the host confers a greater barrier to the development of viral resistance. Currently, LASAG (lysine acetyl salicylate glycine) is the first molecule targeting host intracellular proteins (NF-κB) that is undergoing phase II clinical trials for the treatment of severe influenza virus infection [75]. Inhibitors of NF-κB are expected to limit the production of deleterious cytokines during an infection with highly pathogenic influenza viruses [76].

Virus-host PPIs also provide huge potential for the development of antiviral molecules that directly interfere with the VH interactions. Experimental molecules that disrupt VH PPIs have already been investigated for various viruses, and several pharmaceutical and biotechnology companies have projects focusing on the identification and development of drugs against host targets and VH PPIs (Table 3). Alisporivir is one of the most advanced molecules of this kind that has reached phase III trials for anti-HCV therapy, as part of interferon-free treatment combinations in chronic hepatitis C genotype 1 patients (however, the FDA has put the trial on hold to assess a possible side-effect of pancreatitis). Phase II trial recruitments for chronic hepatitis C genotypes 2 and 3 are ongoing [77]. The drug is a non-immunosuppressive derivative of cyclosporin A (CsA) for which the precise mechanism of action against HCV infection was initially unknown [78]. Later, it was shown that CsA disrupts the interaction between cyclophilin A and NS5A through its binding in the peptidyl-prolyl isomerase hydrophobic pocket of cyclophilin A [79,80]. Use of alisporivir also provides a high barrier to the emergence of resistance, with multiple mutations in domain II of NS5A required *in vitro* for HCV to become resistant [81]. Even if interference of VH PPIs by small molecules proves to be effective for specific anti-viral indications, accumulation of further successful examples will be necessary for this approach to have widespread applicability.

**Table 3:** **Biotechnology companies working on the drugs against host targets and virus-host protein-protein interations**

**Company**	**Web site**	**Location**	**Viral application**	**Stage**	**Mode of action**
Inhikibase Therapeutics	www.inhibikase.com	Atlanta (GA, USA)	Polyomaviruses, HCV, HBV, smallpox virus, ebola virus, RSV, rhinovirus	NC	NC
Forge Life Science	www.forgelifescience.com	Doyleston (PA, USA)	JCV, BKV, CMV, seasonal flu	Research	Enhances the innate role of human sirtuins
Ciclofilin Pharmaceuticals	www.ciclofilin.com	San Diego (CA, USA)	HCV/HBV/HIV co-infection	Preclinical	Inhibitors of cyclophilin
Gemmus Pharma	www.gemmuspharma.com	San Francisco (CA, USA)	FLUAV	NC	Agonist of a G protein-coupled receptor
Springbank Pharmaceuticals	www.springbankpharm.com	Milford (MA, USA)	HCV and HBV	Phase 1	Activates RIG1 and NOD2
iTherX	www.itxpharma.com	San Diego (CA, USA)	HCV (liver transplant)	Phase 1	Entry inhibitors
Prosetta Biosciences	www.prosetta.com	San Francisco (CA, USA)	HCV, FLUAV, HIV1, RABV	SAR	Targets viral capsid host protein interaction
OyaGen Inc	www.oyageninc.com	Rochester (NY, USA)	HIV1	Pre-clinical	APOBEC3G activation and Vif-APOBEC3G interaction
Microbiotix	www.microbiotix.com	Worcester (MA, USA)	Ebola virus	Discovery	Targets NPC1-glycoprotein interaction
Enyo Pharma	www.enyopharma.com	Lyon (France)	HBV, FLUAV, Ebola virus	Lead optimization	NC
Scynexis	www.scynexis.com	Durham (NC, USA)	HCV	Phase 2	Inhibitors of cyclophilin
Vectura	www.vectura.com	Germany and UK	Severe FLUAV	Phase 2	Inhibitor of NF-κB
Novartis	www.novartis.com	Multinational	HCV	Phase 2 and 3	Cyclophilin A-NS5A interaction

*Abbreviations*: *BKV* BK virus, *HBV* hepatitis B virus. *HCV* hepatitis C virus, *CMV* cytomegalovirus, *FLUAV* influenza A virus, *JCV* John Cunningham virus, *NC* not communicated; RABV, rabies virus; RSV, respiratory syncytial virus.

To date, no molecule targeting an intracellular host protein is FDA approved for an antiviral indication. Thus, whether such drugs are truly suitable for the treatment of viral infections remains an open question, mostly because of potential side effects. Nevertheless, it is worth noting that the conventional antiviral compounds are actually quite toxic. Moreover, the duration of the treatment, mostly for acute infections such as with influenza viruses, is not expected to exceed a few days, and this could moderate the incidence of side effects and their severity.

### Drug repositioning

The discovery of new antivirals can be accelerated and rationalized by integrating VH interactomes and drug-related databases. A VH PPI repertoire is indicative of the cellular proteins that are essential for the replication of a given virus. Therefore, these cellular proteins can be considered as potential therapeutic targets whose function could be manipulated by existing small molecules to prevent viral usage and interfere with viral replication. Such modulators of cellular functions, either approved by government authorities or in clinical development for other indications, could be repositioned as new antiviral agents [4,6-9].

Databases that collect information on bioactive small molecules and their protein targets are numerous and differ mainly in their focus and detail level (Table 2). A first comparison of these resources highlights that they are both specific and complementary [82]. However, their standardization in terms of targets and most of all in terms of chemical entities remains a crucial challenge [83]. A preliminary attempt to aggregate several drug-gene interaction resources is available in the drug-gene interaction database (DGIdb), a database that allows the exploration of the human druggable genome [84].

Combining the evolving VH PPI dataset with the drug-target interactions described in DrugBank has already revealed the great potential of drug repurposing for the discovery of antiviral molecules (Figure 3b). This potential has been accelerating since the first high-throughput screenings for VH PPIs.

## Conclusions and perspectives

Since 2007, high-throughput technologies have been applied to VH interactomes, and the number of PPIs and human targets has been growing exponentially ever since. Overall, this new dataset paves the way for the comprehensive understanding of virus life-cycles and host-cell responses. It also opens new horizons for the discovery of host-oriented drugs, whereas most of the antiviral molecules developed so far have only targeted viral components. Basic and pharmaceutical research is now moving towards the targeting of host proteins. Successful examples include the FDA-approved Maraviroc for the treatment of HIV infection, and promising results, for example, for influenza (LASAG, phase II clinical trial, Vectura, Chippenham, UK) and hepatitis C (Alisporivir, phase II clinical trial, Novartis, Basel, Switzerland). These pioneering studies have also demonstrated a reduction in the rate of emergence of antiviral resistance. The explosion in the number of potential targets owing to the recent use of high-throughput technologies has also resulted in an explosion in the number of antiviral drug candidates through the use of repositioning strategies for existing drugs and experimental molecules.

Virus-host interactomes are far from complete and would greatly benefit from the diversification of protein-interaction detection methods to allow the comprehensive exploration of the interactome space. Another major concern is the quality and completeness of the human interactome itself, which is important for prioritizing targets and for proposing strategies of drug combinations based on network pharmacology.

Viruses have evolved with their hosts to manipulate numerous cellular functions, and much can also be learnt from them to control cellular functions that are impaired in non-infectious pathologies. For instance, bioenergetic metabolism plays a pivotal role in the replication of viruses, and the targeting of metabolism by viral proteins can translate into clinical symptoms, best exemplified by chronic hepatitis C, which is characterized by metabolic dysfunction, including insulin resistance. Interestingly, the activity of hexokinase, the first rate-limiting enzyme of glycolysis, is increased upon its interaction with a HCV protein [85]. Mimicking the mechanisms by which this viral protein controls the first step of glycolysis should make it possible to develop novel therapeutic strategies to potentiate glycolysis in metabolic diseases. Testing the hypothesis that genomic mutations and tumor viruses might cause cancer through related mechanisms, Rozenblatt-Rosen and colleagues [13] showed that the analysis of the cellular targets of tumor virus proteins can identify cancer genes with a good success rate. Combined with genomic studies, tumor VH interactomes could therefore become instrumental for the identification of cancer-related genes and proteins and for their prioritization for therapeutic development. These are just two examples from recent studies that indicate that, in addition to paving the way to host-oriented therapeutics for the treatment of viral infections, VH interactomes also have broad implications for the field of non-infectious diseases.

## Abbreviations

CD: Circular dichroism
coAP/MS: Co-affinity purification coupled to mass spectrometry
CsA: Cyclosporin A
FDA: Food and Drug Administration
HBV: Hepatitis B virus
HCV: Hepatitis C virus
HTS: High-throughput screening
HUPO: Human Proteome Organization
IMEx: International Molecular Exchange
LASAG: Lysine acetyl salicylate glycine
ORF: Open reading frame
PPI: Protein-protein interaction
PSI-MI: Proteomics Standards Initiative - Molecular Interaction
SILAC: Stable isotope labeling with amino acid in cell culture
TAP: Tandem affinity purification
VH: Virus-host
Y2H: Yeast two-hybrid
